# Supplementary material for: Significance of wastewater surveillance in detecting the prevalence of SARS-CoV-2 variants and other respiratory viruses in the community – A multi-site evaluation
Source: One Health. 2023 Apr 5;16:100536. doi: 10.1016/j.onehlt.2023.100536 (PMC10074727; doi:10.1016/j.onehlt.2023.100536)
Supplement: Supplementary file 1 — Supplementary Material containing 1) longitudinal genomic RNA analysis of SARS-CoV-2 nucleocapsid genes, N1 and N2, and 2) expanded SARS-CoV-2 variants detection at collection sites. [file mmc1.docx]

**SUPPLEMENTAL INFORMATION**

**
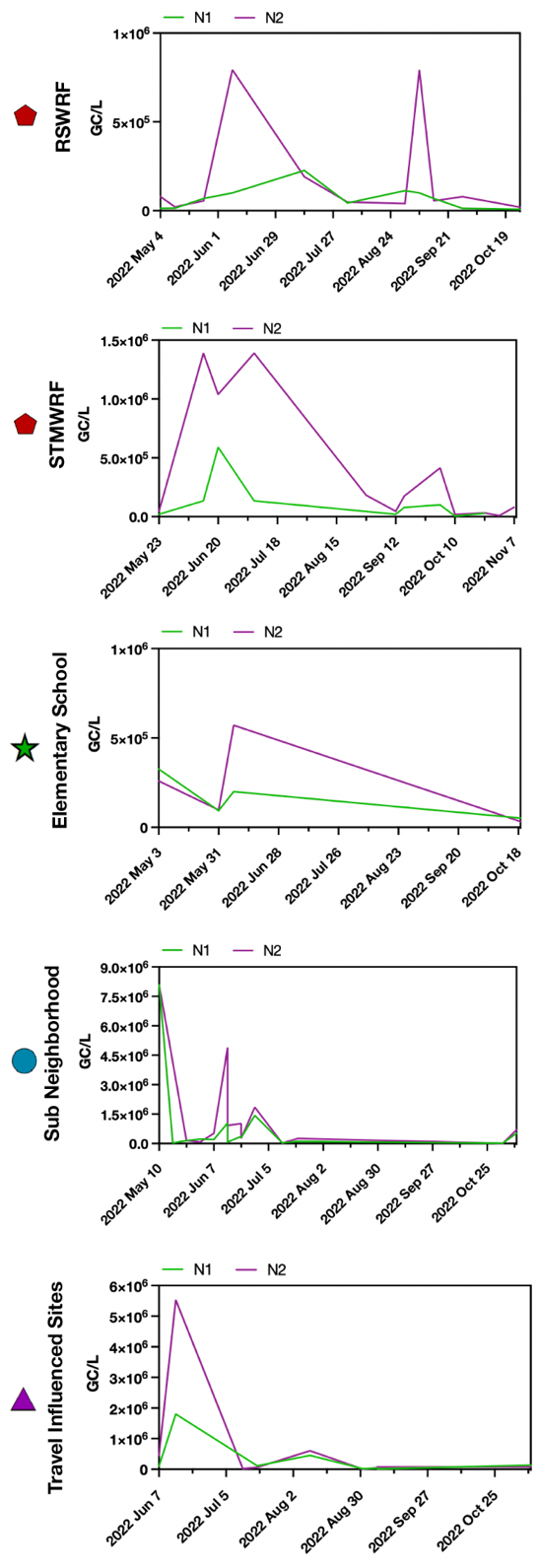
**

**Supplemental Figure 1. Longitudinal Analysis of Genomic RNA of SARS-CoV-2 Nucleocapsid Genes, N1 and N2, at Sub-Sewershed.** Influent was collected from May 2022 -November 2022. RT-qPCR results for N1 (green) and N2 (purple) are plotted over time, subdivided by respective sewershed or WRF collection sites.

**
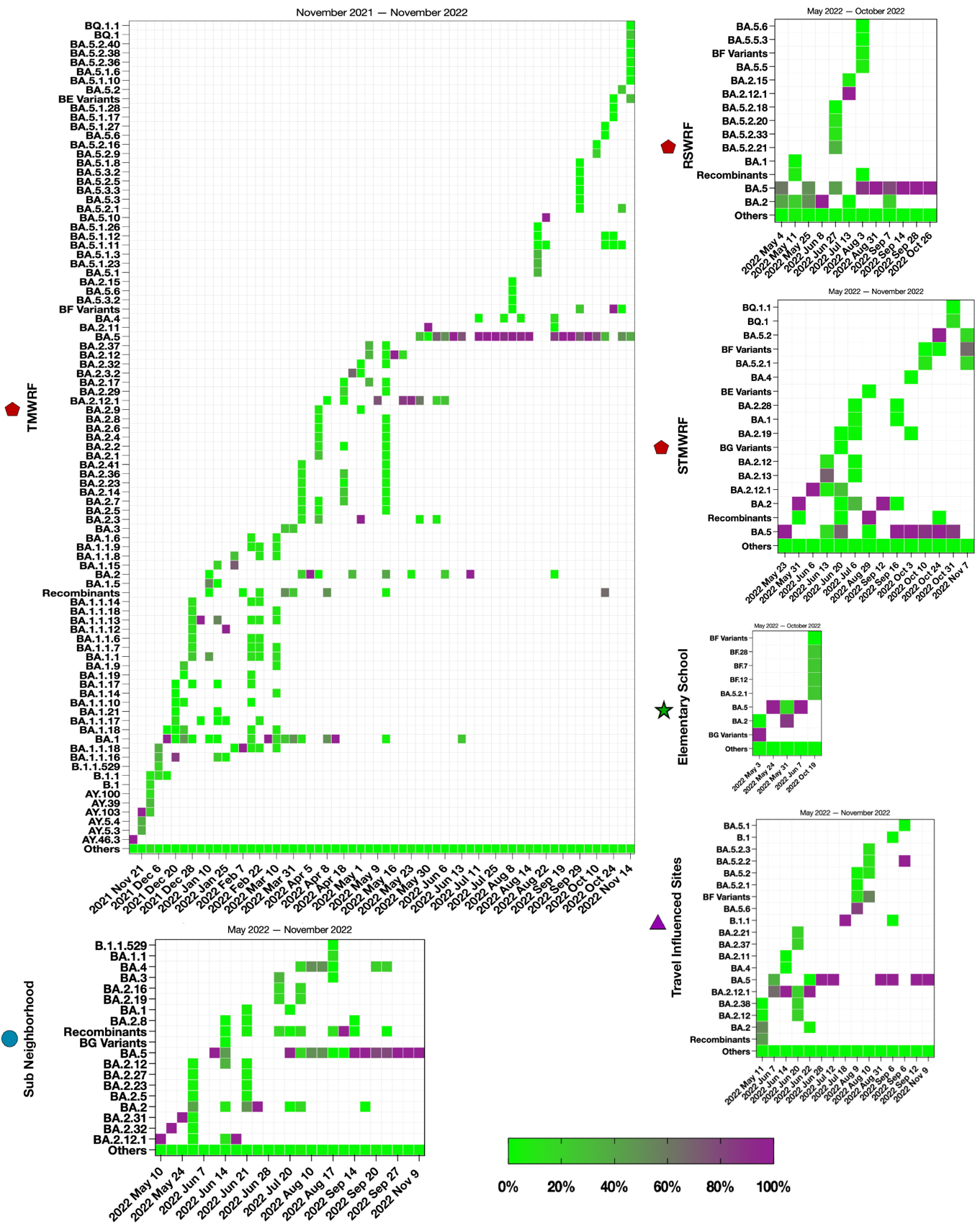
**

**Supplemental Figure 2. Expanded Detection of SARS-CoV-2 Variants in WRFs and Sub-Sewershed.** Influent was collected from November 2022 – November 2022. Collective occurrences of SARS-CoV-2 Delta and Omicron (e.g., AY.X, BA.1.X, BA.2.X, BA.3.X, BA.4.X, BA.5.X, BE.X, BF.X, BG.X, and BQ.X) were calculated. The relative proportion of variants was determined using Freyja, SARS-CoV-2 variants analysis pipeline.
